# Risk for SARS-CoV-2 Infection in Healthcare Workers, Turin, Italy

**DOI:** 10.3201/eid2701.203027

**Published:** 2021-01

**Authors:** Andrea Calcagno, Valeria Ghisetti, Teresa Emanuele, Mattia Trunfio, Silvia Faraoni, Lucio Boglione, Elisa Burdino, Sabrina Audagnotto, Filippo Lipani, Marco Nigra, Antonio D’Avolio, Stefano Bonora, Giovanni Di Perri

**Affiliations:** University of Torino, Torino, Italy (A. Calcagno, M. Trunfio, S. Audagnotto, F. Lipani, A. D’Avolio, S. Bonora, G. Di Perri);; **Azienda Sanitaria Locale** Città di Torino, Torino (V. Ghisetti, T. Emanuele, S. Faraoni, E. Burdino, M. Nigra);; University of Eastern Piedmont, Novara, Italy (L. Boglione)

**Keywords:** severe acute respiratory syndrome coronavirus 2, SARS-CoV-2, coronavirus, viruses, coronavirus disease, COVID-19, respiratory infections, risk, seroprevalence, age, screening, healthcare workers, zoonoses, Turin, Italy

## Abstract

We measured severe acute respiratory syndrome coronavirus 2 spike protein subunits S1/S2 antibodies by using capillary electrophoresis and a chemiluminescence immunoassay for 5,444 active healthcare workers in Italy. Seroprevalence was 6.9% and higher among participants having contact with patients. Seroconversion was not observed in 37/213 previously infected participants.

The ongoing coronavirus disease (COVID-19) pandemic is having an unprecedented impact on the worldwide population. Seroconversion for severe acute respiratory syndrome coronavirus 2 (SARS-CoV-2) was described to occur 7–14 days after onset of symptoms, 100% within 19 days after clinical onset (*1*). Recent serologic data suggest that, in affected areas, SARS-CoV-2 infection had been acquired by more persons than what could be extrapolated by PCR analysis of nasopharyngeal swab specimens (*1**–**3*).

Large studies reported seroprevalences of 1%–6.9% (*2*). In February 2020, seroprevalence for 12 blood donors in Lodi, Italy, a heavily affected zone, was as high as 23% (*3*). Studying high-risk persons, such as healthcare workers, could be relevant for implementing preemptive and protective strategies. In Italy, 30,383 healthcare workers (of 253,619 confirmed cases; 12.0%) have been reported to be infected since the beginning of the pandemic (*4*).

Active healthcare workers (n = 7,457) from **Azienda Sanitaria Locale** Città di Torino public hospitals and outpatient services (Turin, Italy) were invited by email and printed leaflets to participate in our study. During April 17–May 20, 2020, they underwent blood withdrawal. SARS-CoV-2 antibodies were measured by using capillary electrophoresis and chemiluminescence immunoassay targeting IgGs against S1/S2 regions of spike protein (LIAISON; DiaSorin, https://www.diasorin.com). This assay has a sensitivity of 97.9% and a specificity of 98.5% and a 94.4% positive agreement with the plaque reduction neutralization test (*5*). SARS-CoV-2 IgG concentrations were expressed in arbitrary units/mL (AU/mL) and deemed negative if <12 AU/mL. Persons who had equivocal (12–15 AU/mL) or positive (>15 AU/mL) results provided nasopharyngeal swab specimens for SARS-CoV-2 RNA detection by using an in-house real-time reverse transcription PCR, according to Corman et al. (*6*).

Ethics approval was obtained, and all participants signed an informed consent form. Anonymous data were collected and analyzed by using SPSS Statistics version 26 (IBM, https://www.ibm.com) and described as number (%) or mean ± SD. Disease severity information was not collected.

We tested 5,444 (73.0%) of 7,457 healthcare workers; 4,068 (74.7%) were women. Participants had a mean ± SD age of 49.4 ± 10.6 years. S1/S2 SARS-CoV-2 antibodies were found in 377 (6.9%) participants; 176 (46.7%) had cured COVID-19, 146 (38.7%) had contacts with COVID-19 patients, and 55 (14.6%) had no known epidemiologic link. Seroprevalence was not significantly higher in men than in women (7.9% vs. 6.5%; p = 0.097 by χ^2^ test), and no differences were observed among age groups. Mean ± SD IgG titer was 49.2 ± 39.5 AU/mL. IgG titers were higher in older participants (Pearson r = 0.227, p<0.001 and p = 0.001 by analysis of variance; Appendix Figure) and in those previously given a diagnosis of COVID-19 (57.9 AU/mL vs. 41.6 AU/mL in those without a previous diagnosis; p<0.001 by *t*-test).

Detailed task information was available for 4,630 participants. Seroprevalence was highest in laboratory personnel (18/175, 10.3%), although numbers were small, followed by nurse assistants (44/520, 8.5%), nurses (150/1983, 7.6%), and doctors (55/755, 7.3%). A significantly higher seroprevalence was observed in healthcare workers working in close contact with patients versus those with limited/indirect contacts (7.5% vs. 5.2%; p = 0.013 by χ^2^ test; odds ratio 1.464, 95% CI 1.077–1.992) (Figure).

**Figure:**
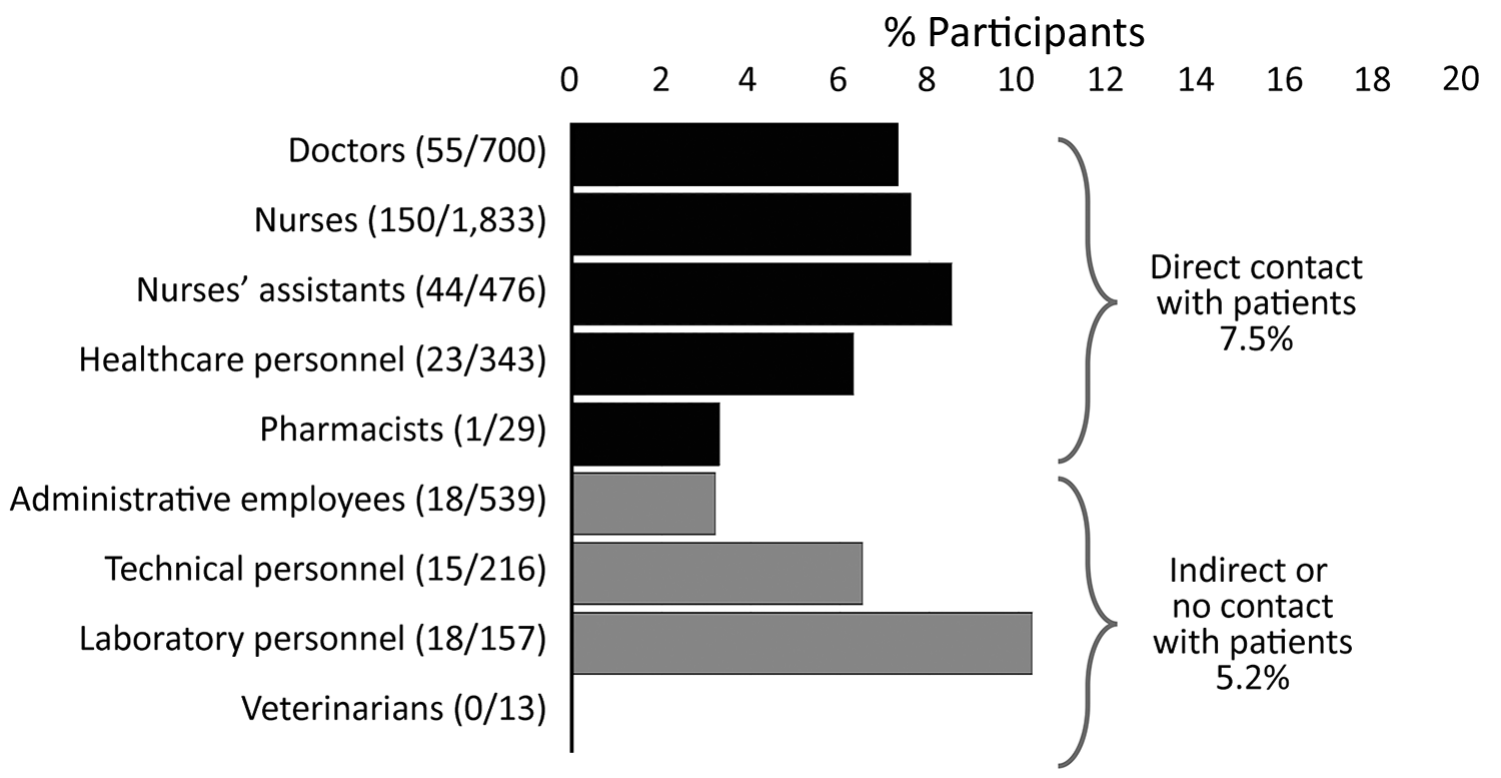
Seroprevalence of severe acute respiratory syndrome coronavirus 2 antibodies in healthcare workers according to tasks of participants, Turin, Italy. Participants are grouped according to direct (black bars) or indirect/no contact (gray bars) with patients. The difference between these 2 groups (7.5% vs. 5.2%) is significant (p = 0.013 by χ^2^ test). The healthcare personnel category includes psychologists, nutritionists, welfare workers, religious assistants, physical therapists, and orthoptists.

Among persons who had a previously diagnosed SARS-CoV-2 infection, 176 (82.6%) had S1/S2 SARS-CoV-2 antibodies. Participants without S1/S2 SARS-CoV-2 antibodies were younger (41.4 vs. 49.1 years; p<0.001) and had a shorter time since diagnosis (36 vs. 44 days; p = 0.008). When we excluded persons who previously had COVID-19, all serology-positive participants (n = 201) provided a nasopharyngeal swab specimen for detection of SARS-CoV-2 RNA; 7 (3.5%) were positive.

We found that SARS-CoV-2 infection had been acquired by 6.9% of healthcare workers in Torino, Italy. Variable seroprevalence has been described among healthcare workers in Belgium (*7*), Spain (*8*), and Germany (*9*) (1.6%–9.3%): no major difference in IgG prevalence was found according to job types. In our study, the highest prevalence was observed for healthcare workers in direct contact with patients and the lowest for administrative staff members. S1/S2 IgG titers were higher in older participants and in those who had a previous diagnosis of COVID-19. In an assay validation study in Boise, Idaho, USA, a seroprevalence of 1.79% was reported; older participants had the highest rates (4%, >80 years of age).

Higher titers in symptomatic patients (we presume were healthcare workers given a diagnosis of COVID-19 according to the local testing policy) have been described (https://www.cdc.gov/coronavirus/2019-ncov/lab/resources/antibody-tests-guidelines.html, https://www.who.int/docs/default-source/coronaviruse/whoinhouseassays.pdf?sfvrsn=de3a76aa_2). Although a shorter time from disease onset might explain the lack of antibodies, a lower seroprevalence in younger, previously infected healthcare workers was unexpected. A total of 3.5% of seropositive participants with no previous diagnosis of COVID-19 had positive PCR results for nasopharyngeal swab specimens; this finding might represent late-stage infections with low/no infectivity.

Our study has limitations, including incomplete coverage of healthcare workers (27% did not respond) and lack of complete job description and disease severity for all participants. Some persons did not show development of IgG after having COVID-19; thus, our study could have missed a subset of previously infected persons (*10*). Despite limitations, our study provides noteworthy estimates about the differential risk for acquiring SARS-CoV-2 infection by healthcare workers according to their specific job setting in a large occupational survey.

Appendix Additional information on risk of SARS-CoV-2 infection in healthcare workers, Turin, Italy.
