# Supplementary material for: Risk for SARS-CoV-2 Infection in Healthcare Workers, Turin, Italy
Source: Emerg Infect Dis. 2021 Jan;27(1):303–5. doi: 10.3201/eid2701.203027 (PMC7774556; doi:10.3201/eid2701.203027)
Supplement: Appendix — Additional information on risk of SARS-CoV-2 infection in healthcare workers, Turin, Italy. [file 20-3027-Techapp-s1.pdf]

Article DOI: https://doi.org/10.3201/eid2701.203027

Risk for SARS-CoV-2 Infection in Healthcare Workers, Turin, Italy

Appendix

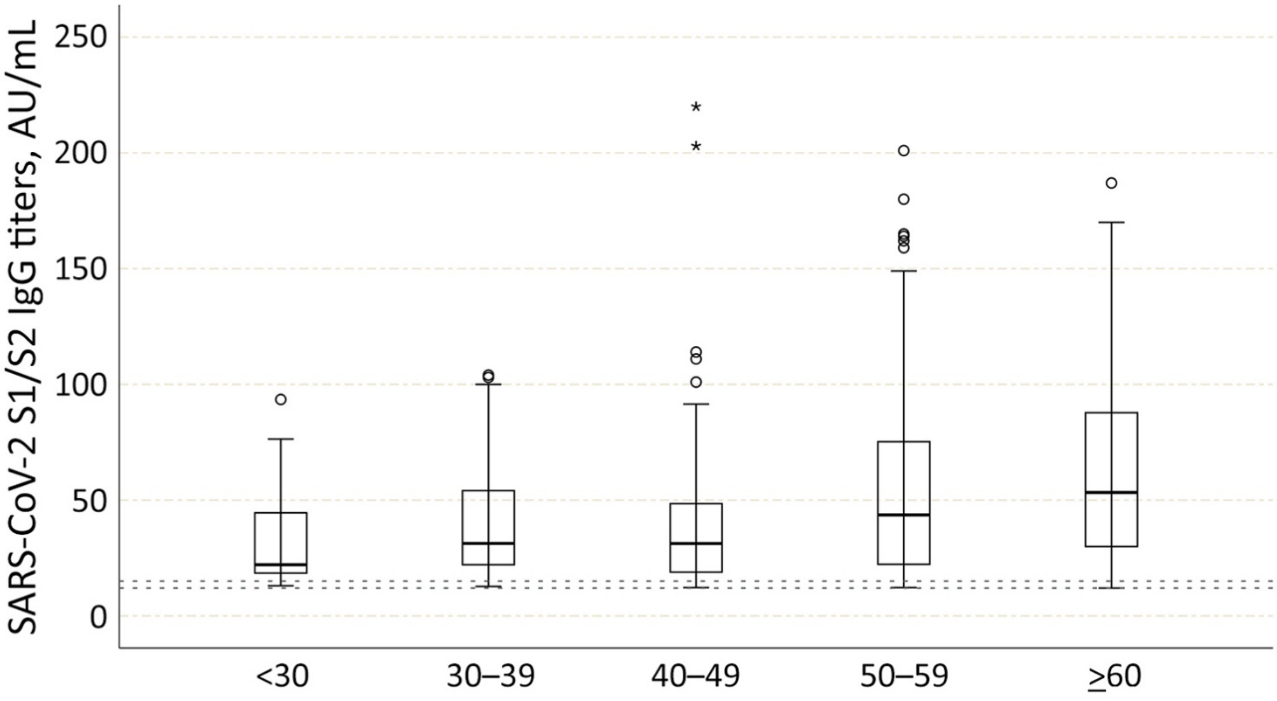

Appendix Figure. Spike protein subunits S1/S2 SARS-CoV-2 antibody arbitrary units according to age for healthcare workers, Turin, Italy. Horizontal bars indicate medians, boxes indicate interquartile ranges, whiskers indicate 10%–90% percentiles, circles indicate outliers, stars indicate extreme outliers, and dashed horizontal lines indicate assay cutoff values (negative: <12 arbitrary units/mL; equivocal: 12–15 arbitrary units/mL; positive: >15 arbitrary units/mL). A statistically significant difference in IgG titers according to age was observed ($p = 0.001$ by analysis of variance). SARS-CoV-2, severe acute respiratory syndrome coronavirus 2.
